# Analytical Performance of the EasyPGX Ready *NTRK* Fusion Assay for Detecting Tumor-Agnostic *NTRK* Gene Fusions in Solid Tumors

**DOI:** 10.3390/cells15201833

**Published:** 2026-10-09

**Authors:** Anna Soiron, Kim Falkenberg, Santina Kirmse, Laura Braun, Elias Dohmen, Daniel Kaschta, Ilka Isfort, Ruth Berthold, Anne Offermann, Eva Wardelmann, Wolfgang Hartmann, Marcel Trautmann

**Affiliations:** 1 Gerhard-Domagk-Institute of Pathology, University Hospital Münster, Domagkstr. 17, 48149 Münster, Germany; 2 West German Cancer Center, University Hospital Münster, 48167 Münster, Germany

**Keywords:** neurotrophic tyrosine receptor kinase, *NTRK1*, *NTRK2*, *NTRK3*, gene fusion, pan-TRK, molecular diagnostics, multiplex RT-PCR, EasyPGX, Diatech, precision oncology

## Abstract

Early detection and accurate characterization of pathogenic *NTRK1*, *NTRK2*, and *NTRK3* gene rearrangements have become essential components of multimodal testing frameworks to select gene fusion-positive candidates for *NTRK*-directed molecularly targeted therapies. This retrospective study presents the first independent validation of the CE-IVD-compliant EasyPGX ready *NTRK* Fusion assay, a ready-to-use RT-PCR workflow for the qualitative detection of *NTRK1*,*2*,*3* gene fusions in solid tumors. Based on the fusion frequency in our institutional pathology cohort, the potential diagnostic coverage of the streamlined fast-track assay was 93.8% (15/16) for *NTRK1*, 100% (1/1) for *NTRK2*, and 58.8% (10/17) for *NTRK3* rearrangement, including *TPM3*, *LMNA*, *TPR*, *STRN*, and *ETV6* as 5′ fusion partners. Twenty-nine archived FFPE tumor specimens with known *NTRK1*,*2*,*3* gene rearrangement status, and two multiplexed FFPE *NTRK* fusion reference standards were tested. Targeted RNA-based next-generation sequencing served as the diagnostic benchmark reference method. Conclusive results were obtained for all 31 RNA samples, including FFPE tumor specimens archived for up to six years. The observed overall sensitivity was 96% (23/24; 95% CI, 78.9–99.9%) and no false-positive results were observed for any of the nine gene fusion targets (observed specificity 100%); the single discordant result occurred in a fusion-positive sample with borderline transcript abundance at the lower limit of the assay’s dynamic range. Dilution experiments indicated valid detection at 6.25 ng RNA input per reaction for all fusion transcripts tested, with robust inter-run repeatability and no false-positive results. In this single-center study, the CE-IVD-compliant EasyPGX ready *NTRK* Fusion assay demonstrated analytical performance within its predefined repertoire of *NTRK1*,*2*,*3* rearrangements, consistent with guideline thresholds, supporting its potential as a first-level fast-track component of multimodal pan-cancer diagnostic workflows.

## 1. Introduction

The cornerstones of oncology practice are experiencing a significant transformation with the adoption of tumor-agnostic principles, catalyzed by the recognition that systemic cancer therapies, including cytotoxic chemotherapy, targeted agents, and immunotherapies, can be selected based on specific molecular alterations rather than solely on the anatomical site or histological subtype of the tumor [1]. Such tumor-agnostic approaches focus on actionable molecular drivers and genetic aberrations that are integral to the pathogenesis of diverse malignancies, thereby enabling the application of precisely matched targeted agents that may substantially improve clinical outcomes for persons with rare or refractory cancers [2,3,4,5]. Several tumor-agnostic biomarkers, including microsatellite instability-high (MSI-H), B-raf proto-oncogene (*BRAF*) V600E mutation, rearranged during transfection (*RET*) gene fusions, human epidermal growth factor receptor 2 (HER2) over-expression, tumor mutational burden-high (TMB-H), and neurotrophic receptor tyrosine kinase (*NTRK*) gene fusions [6,7,8,9,10], have already been integrated into clinical practice, along with the corresponding U.S. Food and Drug Administration (FDA)-approved molecularly targeted therapies [1]. The clinical impact of such milestone approaches in the era of biomarker-driven precision oncology is highlighted by significantly improved clinical outcomes, higher response rates, prolonged median progression-free survival, and extended overall survival compared with non-biomarker-driven strategies [11,12], and continues to shape foundational pan-cancer diagnostic algorithms.

### 1.1. Neurotrophic Tyrosine Receptor Kinase (NTRK) Gene Fusions as Biomarker for Molecularly Targeted Therapy

The *NTRK* gene family comprises three members (*NTRK1*/1q23.1, *NTRK2*/9q21.33, and *NTRK3*/15q25.3), which encode the tropomyosin receptor kinase (TRK) A, B, and C proteins [13]. Physiologically, the TRK signal transduction pathway is essential for the development and function of the nervous system [14,15,16]. In cancer, chromosomal rearrangements generating chimeric *NTRK1*,*2*,*3* gene fusions with diverse 5′ partners result in constitutive, ligand-independent activation of the intrinsic tyrosine kinase domains, thereby driving tumorigenesis by promoting aberrant cell proliferation and survival [16,17,18]. Oncogenic *NTRK1*,*2*,*3* gene fusions have been identified as pathogenic hallmarks and diagnostic biomarkers across a heterogeneous spectrum of malignancies [9,19,20,21], including (i) at high frequencies (>50%) in rather rare neoplasms: infantile fibrosarcoma, secretory carcinoma of the salivary gland, secretory breast cancer, and congenital mesoblastic nephroma, or (ii) at very low (<1%) to low (1–5%) prevalence in more common tumors: colon cancer, melanoma, cholangiocarcinoma, pancreatic cancer, non-small cell lung cancer, breast cancer, glioma, salivary glands neoplasms, thyroid cancer, soft tissue sarcoma, and neuroendocrine tumors. Numerous 5′ fusion partners have been described, with *ETV6::NTRK3* being the most frequent chimeric gene fusion in children and adults, followed by *TPM3::NTRK1* and *LMNA::NTRK1* [9,21].

Given their potent transforming activity during pathogenesis, oncogenic TRK fusion proteins constitute the second tumor-agnostic biomarker class for which several first-generation TRK inhibitors (such as larotrectinib or entrectinib) and second-generation multi-kinase ROS1/TRK inhibitors (such as repotrectinib) have received regulatory FDA approval for patients with locally advanced or metastatic *NTRK*-rearranged solid tumors [22,23,24,25,26,27]. Nonetheless, clinical implementation is challenged by the diverse spectrum of rare *NTRK1*,*2*,*3* gene fusions and the emergence of resistance mechanisms that can limit the effectiveness of TRK inhibitors [17,28]. Therefore, ongoing clinical trials and molecular profiling efforts are essential to expand the selection of precisely matched targeted agents for patients with *NTRK1*,*2*,*3* gene fusion-driven malignancies. Consequently, early identification and accurate characterization of pathogenic *NTRK1*,*2*,*3* gene rearrangements have become essential components of precision oncology programs for selecting appropriate gene fusion-positive candidates for *NTRK*-directed tumor-agnostic therapies [29].

### 1.2. Laboratory Methods for Detecting NTRK1,2,3 Gene Fusions and Their Limitations

Several methods are available for detecting chromosomal *NTRK1*,*2*,*3* rearrangements, including immunohistochemistry (IHC), fluorescence in situ hybridization (FISH), reverse transcriptase polymerase chain reaction (RT-PCR), and next-generation sequencing (NGS). Each of these laboratory techniques has specific advantages and limitations relevant to molecular pathology workflows [19,29,30,31,32].

In routine pan-cancer diagnostic practice, the optimal detection of chimeric *NTRK1*,*2*,*3* gene fusion transcripts often requires a multimodal testing algorithm tailored to the clinical context, tumor histology, available resources, and testing expertise to guide tumor-agnostic anti-*NTRK* treatment strategies with selective TRK inhibitors: (i) initial screening by an efficient and rapid first-level pan-TRK IHC, fast-track RT-PCR, or FISH test in tumor types known to express highly recurrent *NTRK1*,*2*,*3* rearrangements, followed by (ii) confirmatory RNA-based NGS detection for less frequent or novel *NTRK1*,*2*,*3* gene fusion transcripts balancing cost-effectiveness with diagnostic accuracy [29,33,34].

**Targeted RNA-based NGS** is widely regarded as the most comprehensive and sensitive gold standard approach for detecting chimeric *NTRK1*,*2*,*3* gene fusion transcripts across a heterogeneous spectrum of malignancies [29,35,36,37,38,39]. It can detect rare fusion partners and novel transcriptionally active fusion variants, even in limited quantities of tumor tissue. However, the routine implementation of targeted RNA-based NGS is limited by the highly variable RNA quality and/or quantity extracted from formalin-fixed, paraffin-embedded (FFPE) tumor specimens, extended turnaround times, high costs, bioinformatic capabilities, and limited sequencing accessibility in some healthcare settings. DNA-based NGS approaches can also detect *NTRK1*,*2*,*3* gene fusion transcripts, but are less informative regarding functional in-frame RNA transcript architectures and may lack sensitivity compared with RNA-based assays due to challenges in capturing breakpoints within large intronic regions, which may result in false-negative findings [40,41,42].

**Immunohistochemistry** (IHC) using pan-TRK antibodies is a widely used, rapid, and cost-effective screening method that benefits from its broad availability [43,44]. It detects actual TRK protein expression as a surrogate for underlying chromosomal *NTRK1*,*2*,*3* rearrangements but is not informative regarding the functional in-frame RNA transcript architectures. IHC is limited by (i) false positives due to wild-type TRK expression especially in neoplasms showing neural or myogenic differentiation, (ii) false negatives in tumors with low or aberrant protein expression, and (iii) interpretive variability related to staining patterns, intensity, subcellular localization, and potential background artifacts. Additionally, IHC often has a lower sensitivity/specificity (due to variable antibody use and IHC methodology) than RNA-based methods, as it may miss complex chromosomal rearrangements and does not differentiate between the variable spectrum of potential fusion partners. Overall, IHC is an inconclusive screening method that requires subsequent confirmation using a secondary orthogonal method [45,46].

**Fluorescence in situ hybridization** (FISH) is a robust diagnostic method in pathology laboratories for detecting chromosomal rearrangements at the DNA level by visualizing dual-color break-apart signals, indicative of chimeric *NTRK1*,*2*,*3* gene fusions. It is highly sensitive and widely used, particularly for tumor types with a high frequency of recurrent fusion transcripts and known partners (e.g., *ETV6::NTRK3* in salivary gland tumors) [47]. However, break-apart FISH may miss complex or cryptic rearrangements, as it is negatively affected by the genomic distance between the FISH probes on the target chromosome [48]. To determine the fusion status of all three *NTRK1*,*2*,*3* genes, three separate FISH assays must be performed [30]. Furthermore, break-apart FISH does not provide specific information on novel fusion partners, functional in-frame RNA transcript architecture, and oncogenic fusion proteins. Moreover, it requires intact nuclei and expertise for borderline interpretation.

**Reverse transcriptase polymerase chain reaction** (RT-PCR)-based approaches enable the highly sensitive and specific detection of recurrent fusion transcripts with short turnaround times; however, they are intrinsically restricted by the targeted primer design. Because *NTRK1*,*2*,*3* gene fusions involve a wide range of 5′ partners and exon::exon breakpoint architectures (*NTRK1*-HGNC: 8031, ENST00000392302.2, exons: 17; *NTRK2*-HGNC: 8032, ENST00000376214.1, exons: 21; *NTRK3*-HGNC: 8033, ENST00000394480.2, exons: 19), any targeted RT-PCR design inevitably captures only a predefined subset of rearrangements [19]. Therefore, characterizing its diagnostic coverage, defined as the fraction of currently known *NTRK1*,*2*,*3* gene fusion transcripts that are theoretically detectable by a given assay, is essential for interpreting negative results and implementing multimodal pan-cancer testing strategies. Additionally, the analytical sensitivity is adversely affected by RNA quantity and integrity, which are frequently compromised in FFPE tumor samples due to fixation-induced fragmentation, cross-linking, and chemical modification of nucleic acids [49,50]. RT-PCR is frequently used in combination with a secondary orthogonal method as part of standard confirmatory practice.

The **EasyPGX ready *NTRK* Fusion assay** is a CE-IVD-compliant, one-step real-time multiplex RT-PCR kit (RT035, Diatech Pharmacogenetics) intended for the qualitative detection of predefined *NTRK1*, *NTRK2*, and *NTRK3* gene fusion transcripts at the RNA level and does not assess single-nucleotide variants, small insertions/deletions, or other non-fusion sequence alterations. This assay offers a rapid turnaround time (approximately 3 h) and minimal hands-on processing, rendering it suitable for streamlined fast-track routine pan-cancer diagnostic applications. It employs dry, ready-to-use 8-well strips preloaded with multiplexed primer sets and fluorescent probes, enabling the simultaneous detection of multiple recurrent *NTRK1*,*2*,*3* fusion transcripts alongside (i) positive and negative standards included for the validation of each experimental session and (ii) endogenous internal controls for the automated quality assessment of each reaction well. The molecular details of detectable but partially indistinguishable *NTRK1*,*2*,*3* gene fusions using the EasyPGX ready *NTRK* Fusion kit are summarized in Supplementary Table S1. According to Diatech Pharmacogenetics, the limit of detection (LOD) and analytical sensitivity are defined as the lowest input RNA quantity (2.5 ng/reaction established on non-FFPE reference and synthetic RNA) used for RT-PCR amplification that generated the correct result for *NTRK1*,*2*,*3* gene fusion status (RT035, EasyPGX ready *NTRK* Fusion User manual version 2022/03).

Despite CE-IVD compliance of such RT-PCR-based assays, independent validation data on (i) the analytical spectrum and diagnostic coverage across the expanding repertoire of clinically relevant *NTRK1*,*2*,*3* gene fusions, (ii) the analytical performance, and (iii) the empirical limits of detection inter-run repeatability in FFPE tumor specimens remain limited. To our knowledge, no independent validation of the EasyPGX ready *NTRK* Fusion assay in archived FFPE specimens of solid tumors has been published. Therefore, robust evaluations from pathology laboratories under real-world conditions are required to define their appropriate role within multimodal pan-cancer diagnostic algorithms [51].

In this single-center retrospective study, we evaluated the analytical performance of the EasyPGX ready *NTRK* Fusion assay as a ready-to-use RT-PCR workflow for the qualitative detection of *NTRK1*,*2*,*3* gene fusion transcripts in solid tumors, thereby validating its potential as a standardized fast-track component of routine multimodal pan-cancer diagnostic workflows. We hypothesized that the assay would achieve ≥95% sensitivity and ≥98% specificity within its predefined repertoire of *NTRK1*,*2*,*3* rearrangements (Supplementary Table S1), consistent with performance threshold recommendations for molecular diagnostic assays intended for cancer treatment selection (e.g., AMP, Association for Molecular Pathology; CAP, College of American Pathologists) [52,53]. Specifically, we aimed to (i) delineate the analytical spectrum and potential diagnostic coverage using curated data from the Mitelman Database of Chromosome Aberrations and Gene Fusions in Cancer [54], and *NTRK*-rearranged samples identified in our institutional RNA-Seq screening cohort of solid tumors; and (ii) evaluate the analytical sensitivity, specificity, limit of detection, and inter-run repeatability for the intended tumor-agnostic detection of clinically relevant *NTRK1*,*2*,*3* fusion transcripts using archived FFPE tumor specimens from our institutional pathology archive with known *NTRK1*,*2*,*3* rearrangement status, and two multiplexed FFPE *NTRK* fusion reference standards as commercial end-to-end positive controls. Targeted RNA-based NGS served as the diagnostic benchmark reference method, while IHC and FISH findings were incorporated, where available, to reflect real-world multimodal testing practice.

## 2. Materials and Methods

### 2.1. Institutional Pathology Cohort Selection

In total, 29 formalin-fixed, paraffin-embedded (FFPE) tumor specimens with sufficient tissue were retrospectively selected from the archive of the Gerhard-Domagk-Institute of Pathology (West German Cancer Center, University Hospital Münster, Germany). All tissue samples were obtained during routine diagnostic testing for molecularly actionable gene fusions in persons with cancer. A case-control cohort enriched for *NTRK*-rearranged samples was selected to maximize the number of fusion-positive specimens for LOD and repeatability testing, including 16 *NTRK1*,*2*,*3* gene fusion-positive (5x *NTRK1*- and 11x *NTRK3*-rearranged) and 13 *NTRK1*,*2*,*3* gene fusion-negative samples. All samples were previously tested for chimeric *NTRK1*,*2*,*3* gene fusion status using IHC, FISH, and/or targeted RNA-based NGS. Diagnoses, including the most prevalent *NTRK*-rearranged spindle cell neoplasms or infantile fibrosarcomas, were reviewed by two experienced pathologists (E.W. and W.H.) based on the current World Health Organization criteria (WHO) [55]. The mean tumor cell content of all tumor specimens was at least 30%.

For the diagnostic coverage analysis, we used an institutional series of 931 FFPE tumor specimens, routinely screened for gene fusion transcripts by targeted RNA-Seq as part of pan-cancer diagnostic testing. This series is enriched for solid tumors with clinical, histologic or molecular features suggestive of chromosomal rearrangements, and therefore does not represent an unselected pan-cancer population. “Diagnostic coverage” was defined as the proportion of *NTRK1*,*2*,*3* fusion-positive samples in our institutional pathology cohort that harbored gene fusion transcripts represented among the assay’s predefined repertoire of *NTRK1*,*2*,*3* rearrangements (frequency-weighted coverage). This study design does not reflect real-world prevalence (<1% in most tumor types) and therefore does not permit estimation of the positive predictive value (PPV)/negative predictive value (NPV) in unselected populations.

### 2.2. Multiplexed NTRK1,2,3 Gene Fusion Reference Standards

To evaluate the assay performance, analytical sensitivity/specificity, and LOD for clinically relevant *NTRK1*,*2*,*3* gene fusion transcripts, two verified commercial multiplexed reference FFPE samples were included as comprehensive end-to-end process-positive standards (including pre-analytical RNA extraction). Both samples were derived from cell lines: (i) Seraseq FFPE NTRK Fusion RNA (0710-1031, 10 µm curl, SeraCare, Milford, MA, USA), comprising 15 *NTRK1*,*2*,*3* fusion transcripts, and (ii) Pan-Cancer 6-Fusion panel FFPE RNA (HD834, 15 µm curl, Horizon/Revvity, Discovery, Cambridge, UK), including three *NTRK1*,*2*,*3* gene fusions.

### 2.3. RNA Extraction

Total RNA was extracted using magnetic particle technology with the EZ2 RNA FFPE Kit (959734, Qiagen, Hilden, Germany) on an automated EZ2 Connect MDx instrument (Qiagen) and resuspended in 50 µL of RNase-free water. The extracted total RNA was quantified using a Qubit fluorometer and the Qubit RNA HS Assay kit (Invitrogen, Thermo Fisher Scientific, Carlsbad, CA, USA) according to the manufacturer’s instructions.

### 2.4. EasyPGX Ready NTRK Fusion Kit

Qualitative detection of chimeric *NTRK1*,*2*,*3* gene fusion transcripts was performed using one-step multiplex RT-PCR with the CE-IVD-compliant EasyPGX ready *NTRK* Fusion kit (RT035, Diatech Pharmacogenetics, Jesi, Italy), which was validated for use in a streamlined workflow with the EasyPGX qPCR instrument 96 (RT800-96, Diatech Pharmacogenetics), according to the manufacturer’s instructions. In brief, each real-time RT-PCR reaction (ready-to-use 8-well strips preloaded with dry amplification mix, containing multiplexed primer sets and fluorescent probes labeled with FAM or HEX for the detection of chimeric gene fusion transcripts or endogenous internal controls, respectively) was performed in 25 µL using 6.25–50 ng/reaction of FFPE RNA. The RNA input recommended by the manufacturer for FFPE samples is 25–1000 ng/reaction (and 2.5–250 ng/reaction for fresh or frozen tissue). Serial dilutions of total RNA from fusion-positive samples were used to identify the lowest input RNA quantity used for RT-PCR amplification that generated the correct result for *NTRK1*,*2*,*3* gene fusion status across two independent runs. This design provides an empirical estimate of the lowest successfully tested input RNA quantity, rather than a probabilistic LOD95. Input levels below the recommended FFPE range were tested to define the lowest input RNA quantity under stress conditions representative of low-yield archival FFPE material. Positive (multiplexed *NTRK1*,*2*,*3* gene fusion reference standards) and negative (water provided with the kit) controls were run simultaneously. Analytic specificity was calculated at the assay target transcript level by counting the number of non-corresponding *NTRK1*,*2*,*3* fusion targets that remained negative in all RNA samples and reference standards. Because each gene fusion-positive sample contributes true-negative observations for non-corresponding targets, these transcript level observations are not statistically independent. Two-sided 95% confidence intervals (CIs) for sensitivity and specificity were calculated using the Clopper–Pearson method for binomial proportions. To evaluate inter-run repeatability, RNA samples were tested in two independent runs operated on different days on the identical EasyPGX qPCR instrument using the same kit reagent lot and standard operating procedures. Automated raw data validation and on-premise result analysis interpretation were performed using the dedicated Aria (v1.4, Agilent, Santa Clara, CA, USA) and EasyPGX Analysis (v4.0.8, Diatech Pharmacogenetics) software.

### 2.5. Comparative Methods for Detecting NTRK1,2,3 Gene Fusions

#### 2.5.1. Targeted RNA Sequencing (RNA-Seq)

For all available tumor specimens, fusion transcript detection covering 507 genes frequently associated with oncogenic chromosomal rearrangements in cancer was performed based on hybrid-capture next-generation sequencing (NGS) using the TruSight RNA Fusion Panel (RS-304-1002 and RS-304-1003, Illumina, San Diego, CA, USA) as the diagnostic benchmark reference method. First-strand cDNA synthesis was conducted using random hexamers and ProtoScript II Reverse Transcriptase (M0368, New England Biolabs, Ipswich, MA, USA). All purification and size selection steps were performed using Agencourt AMPure XP magnetic beads (A63881, Beckman Coulter, Brea, CA, USA). Targeted RNA-Seq was operated using a benchtop MiniSeq instrument (FC-420-1002, MiniSeq High Output Reagent Kit 150 cycles, Illumina). Two verified multiplexed reference FFPE samples (0710-1031, SeraCare and HD834, Horizon, Revvity Discovery) were included as isogenic-positive standards for routine performance monitoring and evaluation of the targeted RNA-Seq NGS workflow integrity (pre-analytical RNA extraction, NGS library preparation workflow, and post-analytical bioinformatics). We utilized the Arriba algorithm (v2.4.0, https://github.com/suhrig/arriba; accessed on 31 August 2026) on FASTQ RNA-Seq files to detect gene fusion transcripts and for schematic visualization of the exon::exon breakpoint architecture [56].

#### 2.5.2. Immunohistochemistry (IHC)

The CE-IVD ready-to-use VENTANA pan-TRK assay (clone EPR17341, 790-7026, Roche, Tucson, AZ, USA) [44] was used in selected cases as the primary monoclonal rabbit antibody with the BenchMark ULTRA Autostainer system (VENTANA/Roche, Tucson, AZ, USA). This assay is intended for the simultaneous immunohistochemical detection of the C-terminal region of tropomyosin receptor kinase (TRK) proteins A, B, and C, which is conserved across wild-type and chimeric fusion proteins in FFPE tissue specimens. According to the manufacturer’s instructions, 3 μm whole slide FFPE tissue sections were deparaffinized and pre-treated with Cell Conditioning 1 solution (CC1, 950-124, VENTANA/Roche). Immunoreactions were visualized using the OptiView DAB IHC detection kit (760-700, VENTANA/Roche). Tissue sections were counterstained with hematoxylin (790-2208, VENTANA/Roche) and a bluing solution (760-2037, VENTANA/Roche). The IHC-stained sections were interpreted by two experienced pathologists (E. W. and W. H.). The percentage of stained tumor cells was estimated in 10 high-power microscopic fields (20×, 40× magnification).

#### 2.5.3. Fluorescence In Situ Hybridization (FISH)

Qualitative detection of chromosomal rearrangements involving the human *NTRK1* (1q23.1), *NTRK2* (9q21.33), or *NTRK3* (15q25.3) genes was performed on 1.5 µm whole slide FFPE tumor sections of selected tumors. The ZytoLight SPEC *NTRK1* (Z-2167, PL123), *NTRK2* (Z-2205, PL163) or *NTRK3* (Z-2206, PL164) Dual Color Break Apart Probe sets (all ZytoVision, Germany) composed of ZyGreen (excitation: 503 nm, emission: 528 nm) and ZyOrange (547/572 nm) labeled polynucleotides were used according to the manufacturer’s instructions. Fluorescence signal patterns were counted in at least 100 tumor cell nuclei (63× magnification) by two experienced pathologists (A. O. and W. H.).

## 3. Results

### 3.1. Analytical Spectrum and Diagnostic Coverage of the EasyPGX Ready NTRK Fusion Assay

**Figure 1:**
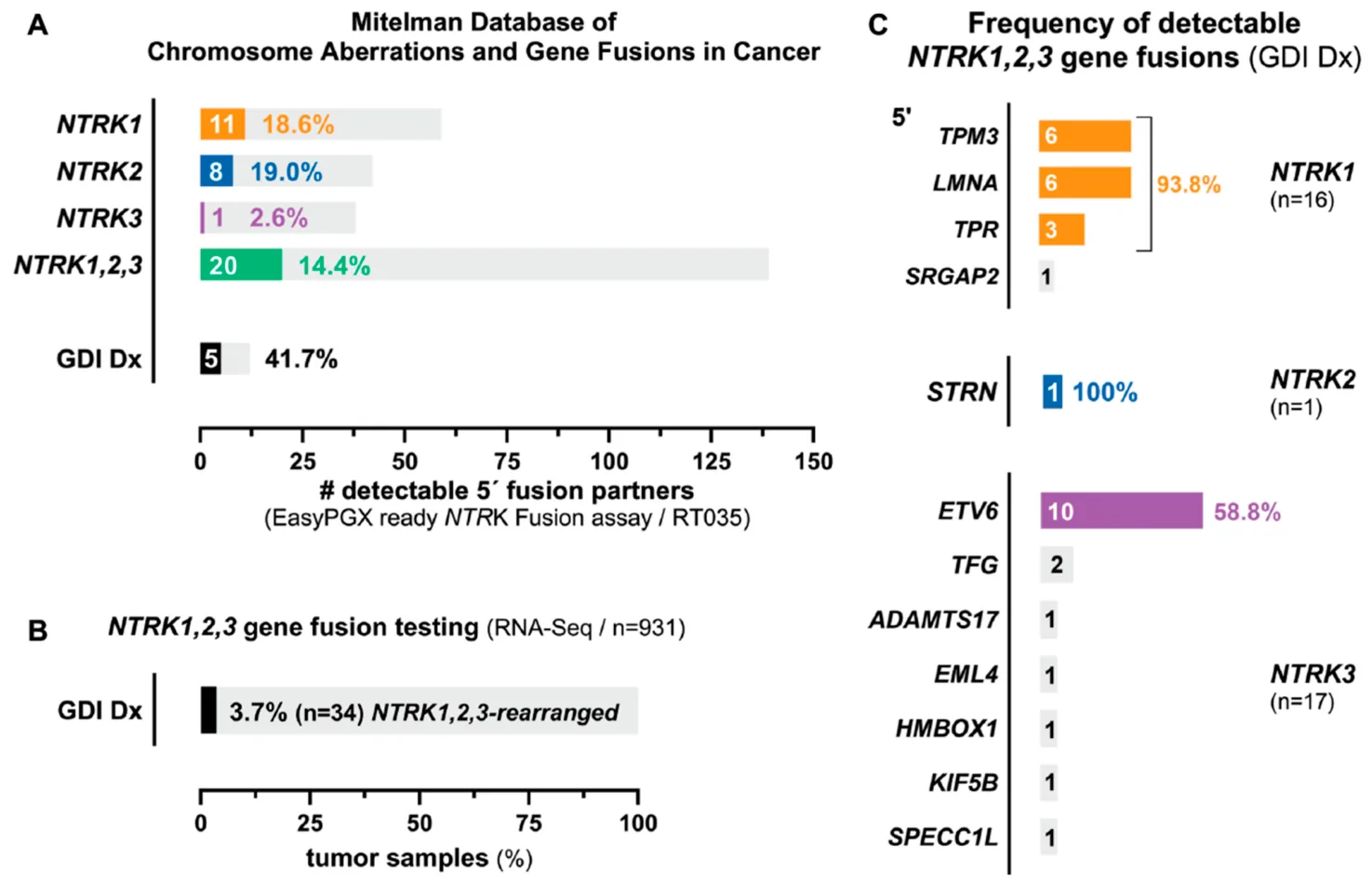
Diagnostic coverage of the EasyPGX ready *NTRK* Fusion assay across reported and institutional *NTRK1*,*2*,*3* gene fusions. (**A**) Distribution of reported *NTRK1* (*n* = 59), *NTRK2* (*n* = 42), and *NTRK3* (*n* = 38) gene fusions in the Mitelman Database of Chromosome Aberrations and Gene Fusions in Cancer (https://mitelmandatabase.isb-cgc.org; last updated on 15 July 2026; in total 34,718 unique gene fusions and 14,130 genes involved). Colored bars indicate the number of fusions potentially covered vs. not covered by the assay. (**B**) *NTRK*-rearranged samples identified by institutional RNA-Seq screening of 931 FFPE solid tumor specimens (34 fusion-positive, 3.7%). (**C**) Frequency-weighted diagnostic coverage, calculated as the proportion of *NTRK1*,*2*,*3* gene fusion-positive samples in the institutional pathology cohort whose specific fusion transcripts are theoretically detectable by the multiplex primer design of the assay (colored bars). Importantly, these center specific estimates are dependent on our institutional pathology cohort and should not be extrapolated to other institutions without considering local tumor epidemiology and diagnostic referral patterns.

In total, 139 structural chromosome aberrations involving *NTRK1* (59), *NTRK2* (42), or *NTRK3* (38) as rearranged 3′ fusion partners have been reported, based on the information in the Mitelman Database of Chromosome Aberrations and Gene Fusions in Cancer (https://mitelmandatabase.isb-cgc.org; last updated on 15 July 2026, comprising 34,718 unique gene fusions and 14,130 genes involved). Overall, the EasyPGX ready *NTRK* Fusion assay design contains multiplexed primers that potentially cover 14.4% (20/139) of the unique *NTRK1*,*2*,*3*-rearranged fusion variants catalogued in the Mitelman Database, irrespective of their reported clinical recurrence, comprising *NTRK1* (18.6%; 11/59), *NTRK2* (19.0%; 8/42), and *NTRK3* (2.6%; 1/38; with *ETV6* as the most frequently reported 5′ fusion partner in both pediatric and adult solid tumors covered) gene fusions (Figure 1A and Supplementary Table S1).

During routine pan-cancer diagnostic screening, we tested 931 RNA samples isolated from FFPE tumor specimens for the expression of chimeric *NTRK1*,*2*,*3* gene fusion transcripts and identified 34 (3.7%) fusion-positive individuals diagnosed with various cancer types (Figure 1B), displaying 12 fusion partners (Figure 1C). Overall, the EasyPGX ready *NTRK* Fusion assay potentially covers 41.7% (5/12) of the *NTRK1*,*2*,*3* rearranged fusion transcripts detected in our institutional pathology cohort of archival FFPE tumor samples, including those with *TPM3*, *LMNA*, and *TPR (NTRK1)*, *STRN (NTRK2)*, and *ETV6 (NTRK3)*, as identified 5′ fusion partners (Figure 1A). Focusing on the frequency of occurrence in our institutional pathology cohort, the diagnostic coverage across the repertoire of clinically relevant *NTRK1*,*2*,*3* gene fusions is 93.8% (15/16) for *NTRK1*, 100% (1/1) for *NTRK2*, and 58.8% (10/17) for *NTRK3* (Figure 1C and Supplementary Table S1).

**Figure 2:**
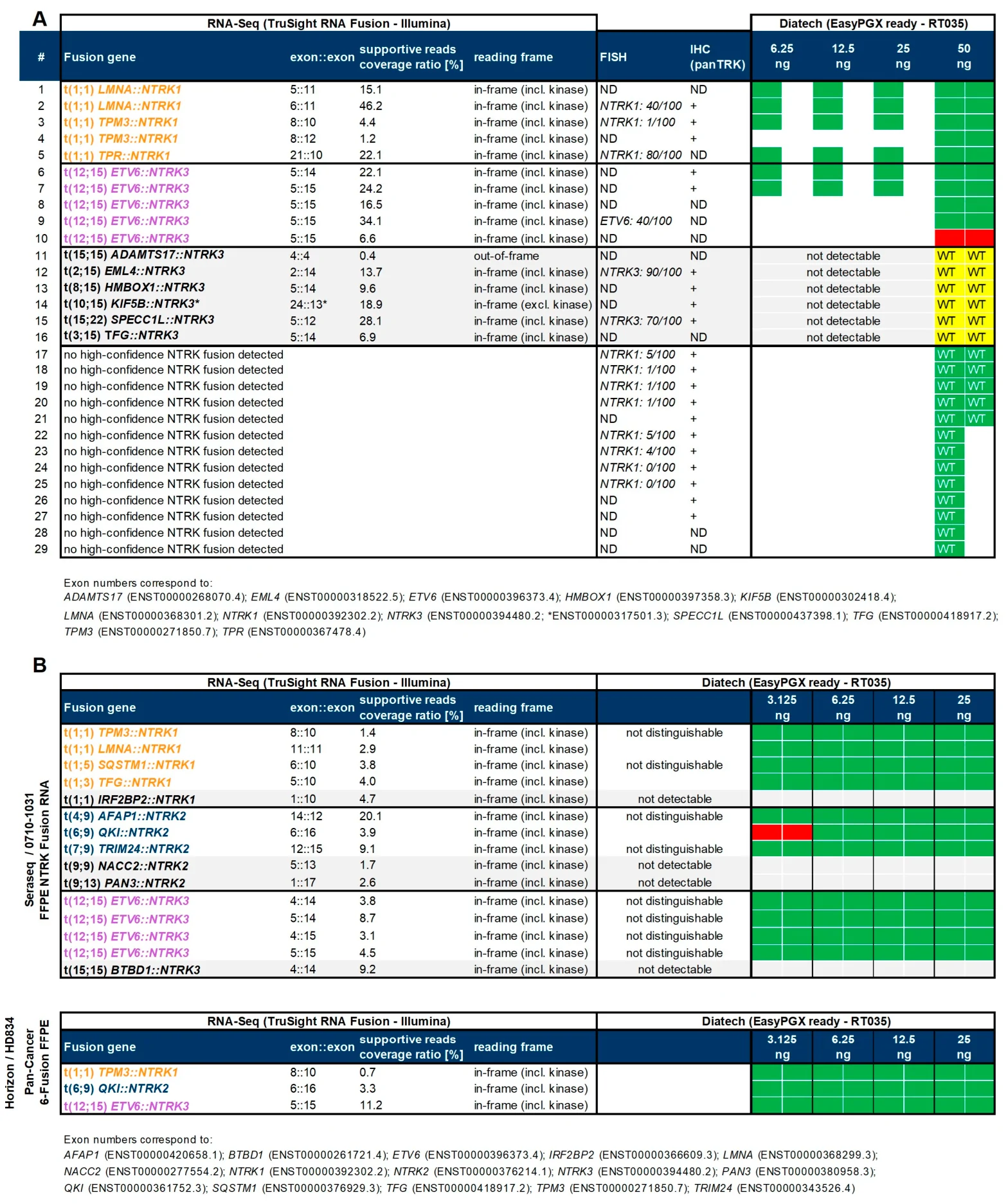
*NTRK1*,*2*,*3* gene rearrangement status and assay concordance for 29 archived FFPE tumor specimens and two commercial multiplexed *NTRK* fusion reference standards. (**A**) Rows represent individual tumor samples (#1–29: institutional pathology archive, median archival time 2 years, range 1–6 years). (**B**) Commercial reference standards: Seraseq FFPE NTRK Fusion RNA (0710-1031, 10 µm curl, SeraCare) comprising 15 *NTRK1*,*2*,*3* fusion transcripts, and Pan-Cancer 6-Fusion panel FFPE RNA (HD834, 15 µm curl, Horizon/Revvity). Columns indicate: Benchmark *NTRK1*,*2*,*3* rearrangement status by targeted RNA-Seq, exon::exon breakpoint architectures, supporting methods (IHC, FISH) where available, EasyPGX ready *NTRK* Fusion assay test results (green: concordant/red: discordant), and RNA input range tested (ng/reaction). “WT”/wild-type (no fusion transcript detected). Grey shading indicates *NTRK1*,*2*,*3* gene fusions not covered by the multiplex primer design of the assay (Supplementary Table S1).

From the archive of the Gerhard-Domagk-Institute of Pathology (West German Cancer Center, University Hospital Münster, Germany), we assembled 16/34 FFPE tumor specimens with positive *NTRK1* or *NTRK3* chromosomal rearrangement status (Figure 2A; sample #1–16), including 10 potentially covered by the multiplex primer design of the EasyPGX ready *NTRK* Fusion assay (sample #1–10). In addition, we selected 13 FFPE samples previously tested negative for high-confidence *NTRK1*,*2*,*3* gene fusion transcripts by targeted RNA-Seq, including samples with challenging borderline pan-TRK IHC/FISH results (Figure 2A; sample #17–29).

### 3.2. Analytic Performance of the EasyPGX Ready NTRK Fusion Assay

All archived FFPE tumor specimens (*n* = 29) available for this retrospective study and two verified commercial multiplexed *NTRK1*,*2*,*3* gene fusion reference standards (Figure 2B) were analyzed using the EasyPGX ready *NTRK* Fusion assay to evaluate the analytical performance of the kit for detecting chimeric *NTRK1*,*2*,*3* gene fusions. The median archival time of all analyzed FFPE tumor specimen blocks was 2 years (range: 1–6 years). Valid test results were obtained for all individual 31 RNA samples in approximately 3 h. RNA sequencing data were available as a benchmark for all RNA samples tested to assess the analytical concordance with our routine pan-cancer diagnostic reference method. The mean percentage of RNA-Seq reads supporting *NTRK1*,*2*,*3* gene fusion is 10.8% (median: 6.8%; range: 0.4–46.2).

**Figure 3:**
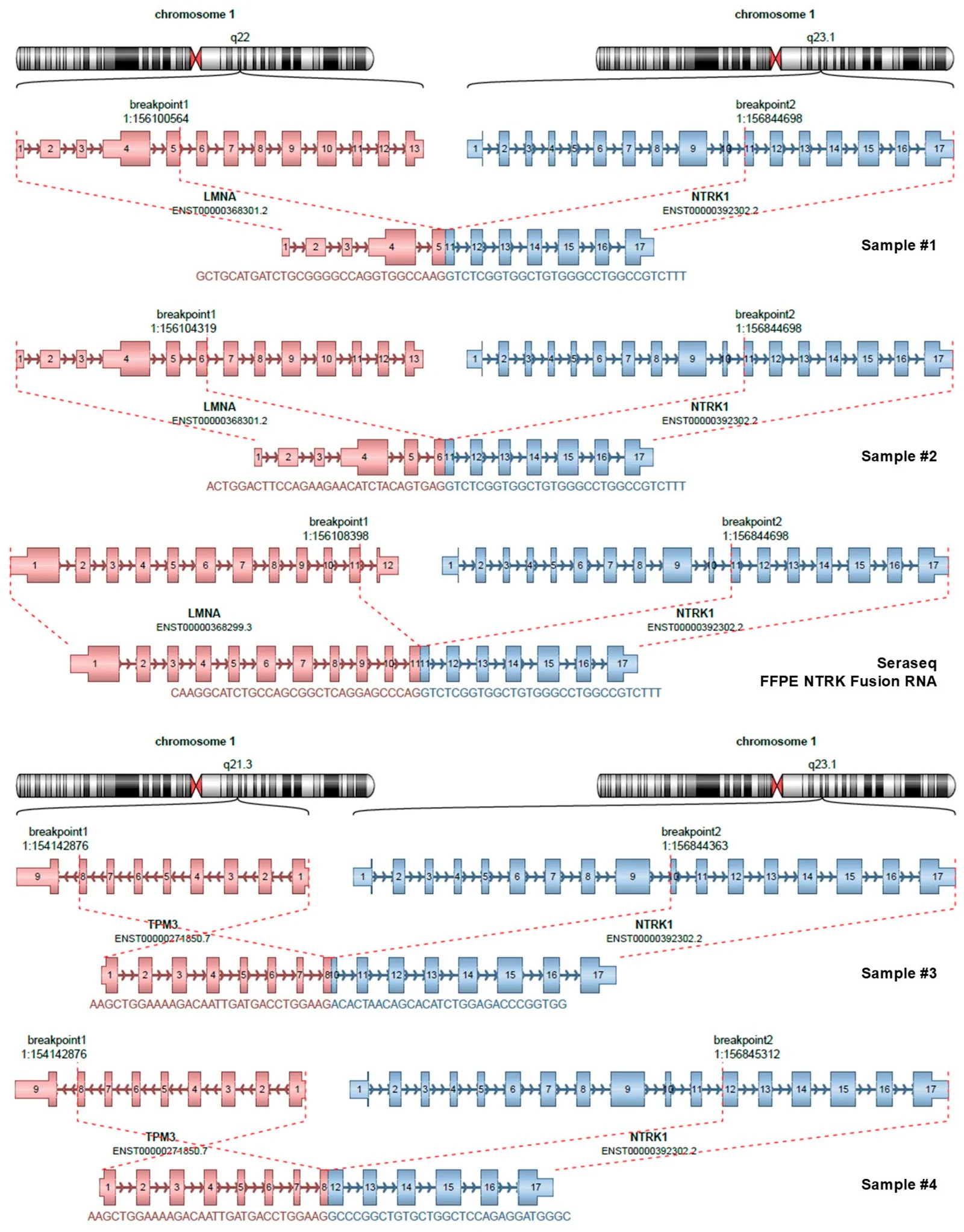

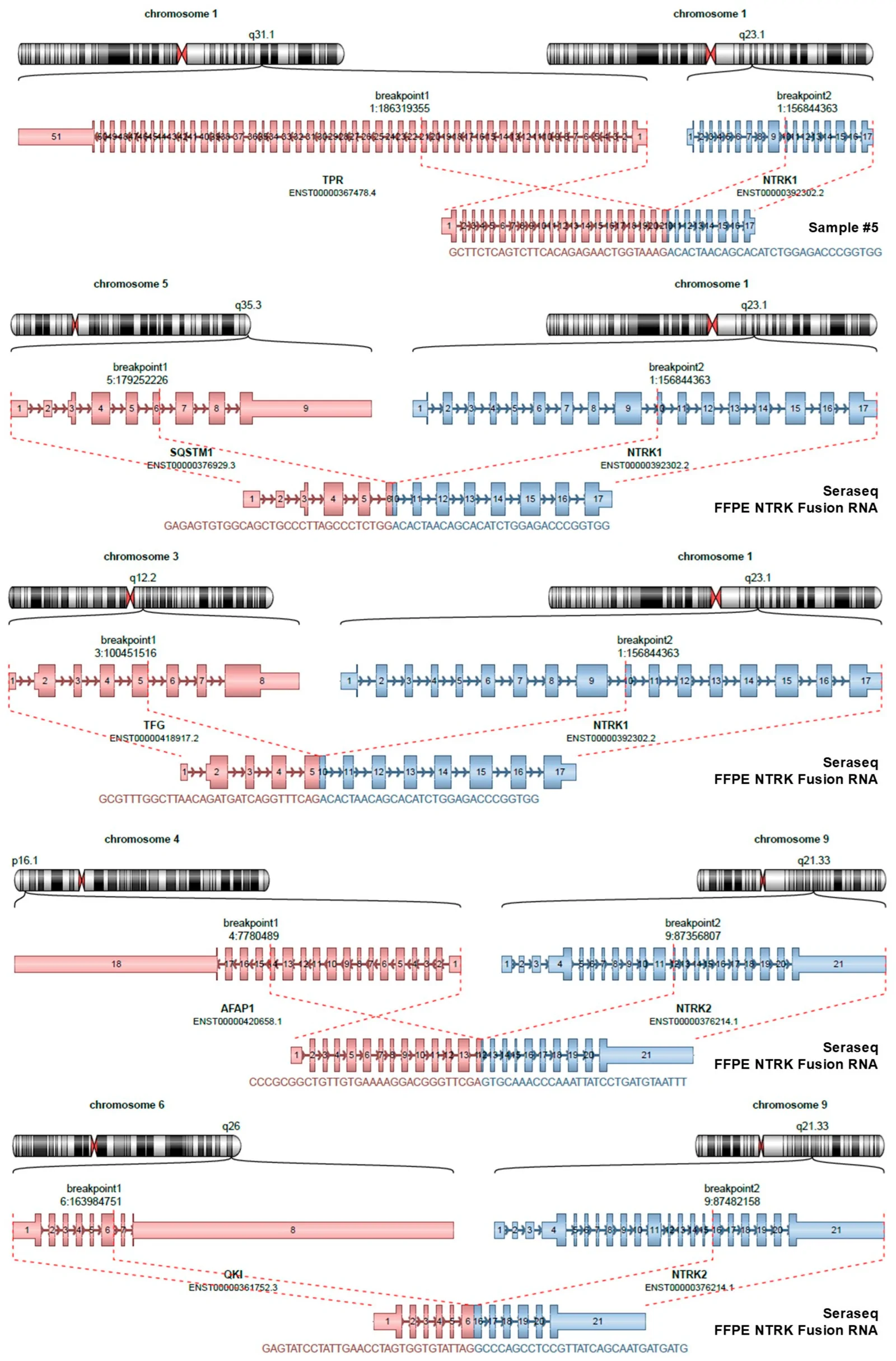

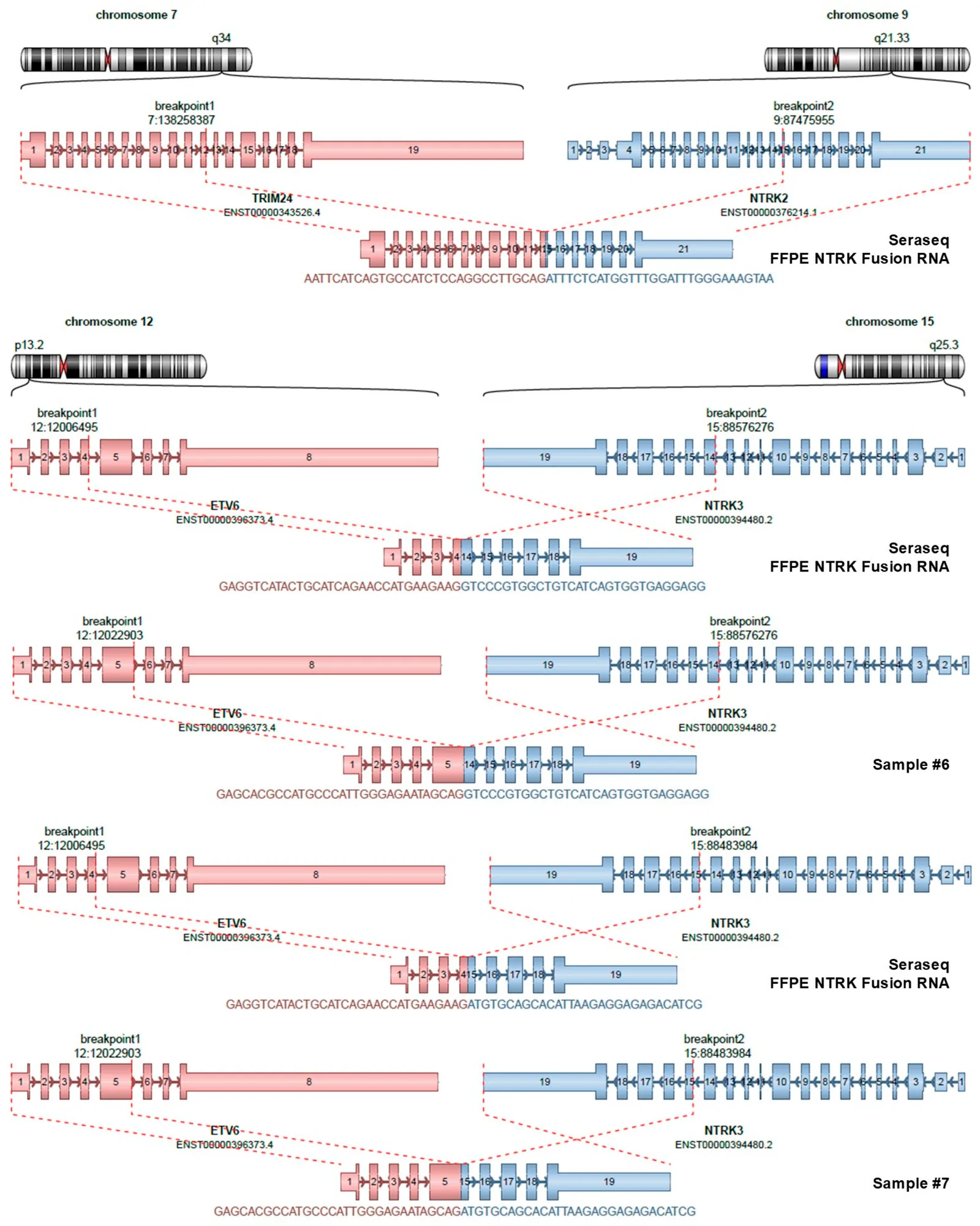
Schematic visualization of exon::exon breakpoint architectures for chimeric *NTRK1*,*2*,*3* gene fusion transcripts correctly detected by the EasyPGX ready *NTRK* Fusion assay. Individual fusion breakpoint junctions with the 5′ fusion partner (left, red) and rearranged 3′ *NTRK* genes (right, blue boxes) are shown. (Arriba algorithm, v2.4.0, https://github.com/suhrig/arriba; accessed on 31 August 2026).

With an RNA input range starting from 50 ng and diluted to 3.125 ng/reaction, a discordant test result was reported by the EasyPGX ready *NTRK* Fusion assay compared to the individual RNA-Seq benchmark results (Figure 2). Even with an input of 50 ng, the in-frame *ETV6::NTRK3* (exon breakpoint junction 5::15) fusion transcript in sample #10 was classified as “WT”/wild-type for the presence of chimeric *NTRK1*,*2*,*3* gene fusion transcripts covered by the assay, which had previously tested positive with 6.6% supportive reads per coverage ratio by targeted RNA-Seq. According to Diatech Pharmacogenetics troubleshooting recommendations, visual inspection of the individual amplification plot with the EasyPGX Analysis (v4.0.8, Diatech Pharmacogenetics) software indicated (i) valid positive and negative control reactions, (ii) sufficient amplifiable RNA sample quantity (endogenous control gene, HEX), and (iii) a correct but weak target amplification fluorescence signal (FAM) above the predefined FAM Cq cut-off value of ≤ 35.0 for the *NTRK3* ex15 amplification mix. An additional discordant result was obtained in a LOD dilution series (3.125–25 ng/reaction) of the multiplexed Seraseq FFPE NTRK Fusion RNA reference standard, which was negative for the in-frame *QKI::NTRK2* (exon breakpoint junction 6::16) fusion transcript with the lowest RNA input of 3.125 ng. All ten *NTRK1*,*2*,*3* gene fusion transcripts not covered by the EasyPGX ready *NTRK* Fusion multiplex primer design were classified as “WT”/wild-type, and no false-positive result was called by the EasyPGX Analysis software. Representative amplification curves generated by the EasyPGX qPCR instrument 96 for (i) *NTRK* fusion-positive (A, #2), (ii) *NTRK* fusion-negative (B, #18), (iii) by the EasyPGX ready *NTRK* Fusion multiplex primer design not detectable (C, #15), and (iv) discordant (D, #10) samples are illustrated in Supplementary Figure S1 A-D. All correctly detected chimeric *NTRK1*,*2*,*3* gene fusion transcripts rearranged to *LMNA*, *TPM3*, *TPR*, *SQSTM1*, *TFG*, *AFAP1*, *QKI*, *TRIM24*, or *ETV6* as 5′ fusion partners are summarized with the specific exon::exon breakpoint junctions shown in Figure 3.

### 3.3. Analytic Sensitivity and Specificity

The global sensitivity (SE) and specificity (SP) of the EasyPGX ready *NTRK* Fusion assay for detecting all tested *NTRK1*,*2*,*3* gene fusion transcripts were 96% (23/24; 95% CI, 78.9–99.9%) and 100% (258/258), respectively, using targeted RNA-Seq as the diagnostic benchmark reference method (Table 1 and Figure 2). The observed point estimates were consistent with the pre-specified targets (≥95% and ≥98%), although the lower bounds of the 95% CIs do not allow formal confirmation given the cohort size. Each positive sample served as a negative control for alternative *NTRK1*,*2*,*3* rearrangements, which are therefore not statistically independent. For *LMNA::NTRK1*, *TPM3::NTRK1*, *TPR::NTRK1*, *SQSTM1::NTRK1*, *TFG::NTRK1*, *AFAP1::NTRK2*, *QKI::NTRK2*, and *TRIM24::NTRK2*, all tested fusion-positive samples were correctly detected, corresponding to sensitivities of 100% (95% CI, 2.5–100.0% to 39.8–100.0%, depending on the number of *NTRK*-rearranged samples). For *ETV6::NTRK3*, nine of ten fusion-positive samples were detected, resulting in a sensitivity of 90.0% (95% CI, 55.5–99.7%). No false-positive results were observed for any of the nine *NTRK1*,*2*,*3* fusion transcripts. Consequently, the observed specificity was 100% for all nine gene fusion targets, with lower bounds of the exact 95% CIs ranging from 85.8% to 88.4% (depending on the number of fusion-negative samples tested). The relatively wide confidence intervals for *NTRK1*,*2*,*3* gene fusion transcripts represented by only one positive sample reflect the limited cohort size.

### 3.4. Limit of Detection (LOD)

The limit of detection for specific *NTRK1*,*2*,*3* rearrangements was defined as the lowest input quantity of RNA used for qPCR amplification, generating the correct *NTRK1*,*2*,*3* gene fusion status for all tested FFPE samples and replicates, rather than a probabilistic LOD95. We assessed the LOD of the EasyPGX ready *NTRK* Fusion assay by diluting the RNA input of (i) six archived FFPE tumor specimens (6.25–50 ng/reaction) and (ii) two verified commercial multiplexed reference FFPE samples (3.125–25 ng/reaction) in two independent runs (Table 2 and Figure 2). Across the dilution series, concordant results demonstrated that using an RNA input of at least 6.25 ng, the specifically tested *NTRK1*,*2*,*3* gene fusion transcripts could be effectively detected.

### 3.5. Repeatability (Inter-Run Variability)

To assess the inter-run repeatability of the system, 23 RNA samples (ranging from 3.125–50 ng/reaction) were tested in two independent runs, performed on different days on the identical EasyPGX qPCR instrument 96 using the same kit reagent lot and standard operating procedures. Under routine workflow conditions, concordant results consistently confirmed the individual *NTRK1*,*2*,*3* rearrangement status for all chimeric gene fusion transcripts and samples tested (Figure 2). No false positives were observed during the repeatability test runs.

## 4. Discussion

The objective of this retrospective study was to evaluate the analytical performance of the CE-IVD-compliant EasyPGX ready *NTRK* Fusion assay as a ready-to-use multiplex RT-PCR-based workflow for the fast-track detection of tumor-agnostic *NTRK1*,*2*,*3* gene fusions in FFPE specimens of solid tumors.

A key observation of this study is the discrepancy between the assay’s modest analytical spectrum and diagnostic coverage (Supplementary Table S1) across the expanding repertoire of clinically relevant *NTRK1*,*2*,*3* rearrangements (14.4%, 20/139) of reported *NTRK1* (18.6%; 11/59), *NTRK2* (19.0%; 8/42), or *NTRK3* (2.6%; 1/38) gene fusions (Figure 1), and the assay’s empirical coverage of clinically observed *NTRK1*,*2*,*3* gene fusions during our institutional pan-cancer diagnostic screening of 931 RNA samples isolated from archived FFPE solid tumor specimens. Systematic reviews and large pan-cancer studies have reported hundreds of distinct *NTRK1*,*2*,*3* fusion partners and exon::exon architectures across solid tumors [8,9,57,58,59], with pronounced heterogeneity outside a focused set of recurrent events, such as *ETV6::NTRK3*, *TPM3::NTRK1*, and *LMNA::NTRK1* rearrangements. In this context, an RT-PCR-based workflow that does not cover the full diversity of *NTRK1*,*2*,*3* rearrangements [45,60] cannot serve as a standalone comprehensive detection method and is therefore best positioned as a first-level fast-track component within a multimodal testing algorithm. However, when benchmarked against our institutional pathology cohort (*n* = 931 FFPE specimens), which is enriched for suspected fusion-positive solid tumors, the EasyPGX ready *NTRK* Fusion assay potentially covers 41.7% (5/12) of the *NTRK1*,*2*,*3* rearranged fusion transcripts, including *TPM3*, *LMNA*, *TPR*, *STRN*, and *ETV6*, as 5′ fusion partners (Figure 1). When weighted by the frequency of occurrence in our institutional pathology cohort, the diagnostic coverage increased to 93.8% (15/16) for *NTRK1*, 100% (1/1) for *NTRK2*, and 58.8% (10/17) for *NTRK3* (Figure 1). This frequency-weighted metric is more clinically relevant than coverage based on unique *NTRK1*,*2*,*3* fusion variants, as it reflects the likelihood that a person with an *NTRK*-rearranged tumor in our institutional pathology cohort would be detected using this assay. Importantly, these center specific estimates should not be extrapolated to other institutions without considering local tumor epidemiology and referral patterns. Overall, this pattern reflects both the non-uniform distribution of *NTRK1*,*2*,*3* gene fusions in clinical practice and the assay manufacturer’s focus on tumor-agnostic testing and the most recurrent 5′ fusion partners, consistent with recommendations that RT-PCR workflows should prioritize guideline-relevant, molecularly actionable gene fusions dedicated to precision oncology [29].

Using targeted RNA-Seq as the diagnostic reference benchmark method, our data indicate that the EasyPGX ready *NTRK* Fusion assay achieved an observed overall sensitivity of 96% (23/24; 95% CI, 78.9–99.9%), with all fusion-positive transcripts detected for eight of nine predefined gene fusion targets and 9/10 for *ETV6::NTRK3* (95% CI, 55.5–99.7%). No false-positive results were detected for any of the nine fusion transcripts covered by the targeted primer design (Table 1 and Figure 2), resulting in an observed specificity of 100% across all tested *NTRK1*,*2*,*3* gene fusions with lower bounds of the exact 95% CIs ranging from 85.8% to 88.4% (depending on the number of fusion-negative samples tested). The relatively wide confidence intervals for *NTRK1*,*2*,*3* gene fusion transcripts represented by only one positive sample reflect the limited cohort size. Hence, under real-world laboratory conditions and within its predefined repertoire of tumor-agnostic *NTRK1*,*2*,*3* rearrangements (Supplementary Table S1), the streamlined EasyPGX ready *NTRK* Fusion workflow attains a level of diagnostic accuracy compatible with previously reported performance evaluations and recommendations for RT-PCR-based *NTRK1*,*2*,*3* gene fusion assays [29,30,61]. Conclusive results were obtained across all archived FFPE tumor specimens (*n* = 29) stored for up to 6 years in our institutional pathology archive, and inter-run repeatability was robust, with consistent gene fusion status across independent runs and RNA input ranges starting from 50 ng and diluted to 3.125 ng/reaction (Table 2 and Figure 2). This is clinically relevant because current guidelines recognize that pre-analytical variability and tissue aging are major challenges in tumor-agnostic gene fusion testing [29,30]. Overall, no false-positive results were called by the EasyPGX Analysis software, with correctly detected chimeric *NTRK1*,*2*,*3* gene fusion transcripts rearranged to *LMNA*, *TPM3*, *TPR*, *SQSTM1*, *TFG*, *AFAP1*, *QKI*, *TRIM24*, and *ETV6* as 5′ fusion partners (summarized with the specific exon::exon breakpoint junctions in Figure 3). Near the lower end of the assay’s dynamic range, discordant test results were reported by the EasyPGX ready *NTRK* Fusion assay (Figure 2) despite adequate internal controls and positive benchmarking by targeted RNA-Seq for: (i) one in-frame *ETV6::NTRK3* (exon breakpoint junction 5::15) gene fusion with borderline transcript abundance (6.6% supportive reads by RNA-Seq, sample #10) was classified as “WT”/wild-type even with an input of 50 ng, and (ii) loss of in-frame *QKI::NTRK2* (exon breakpoint junction 6::16) fusion transcript detection at the lowest RNA input level (LOD dilution series 25 ng to 3.125 ng/reaction) of the multiplexed Seraseq FFPE NTRK Fusion RNA reference standard. For the *ETV6::NTRK3*-positive sample, a correct but weak target-specific amplification signal was generated above the predefined Cq cutoff, consistent with a fusion transcript abundance at the lower end of the assay’s dynamic range. According to the manufacturer’s instructions, such cases are flagged for visual inspection of the amplification plot and reflex confirmatory testing. Our observation supports the caution emphasized in consensus algorithms and, according to Diatech Pharmacogenetics troubleshooting recommendations, that negative test results from RT-PCR-based assays do not exclude *NTRK1*,*2*,*3* gene fusions in samples with high pre-test probability and should prompt (i) visual inspection of the individual amplification plots (Supplementary Figure S1) with the dedicated EasyPGX Analysis software for on-premise use with an EasyPGX ready-to-use kit, and (ii) confirmatory NGS-based testing [29,30]. The discordant *ETV6::NTRK3* sample underscores that increasing total RNA input alone does not guarantee detection when the amount of amplifiable fusion transcript is low. Notably, the same *ETV6::NTRK3* (exon 5::15 breakpoint junction) was correctly detected in three additional clinical specimens (#7–9) and in both reference standards down to 3.125 ng/reaction (Table 2, Figure 2), arguing against a systematic primer/probe design limitation for this specific *ETV6::NTRK3* breakpoint and pointing to sample-specific factors (e.g., transcript abundance or low RNA integrity).

International consensus recommendations and guidelines converge on a multimodal framework for tumor-agnostic *NTRK1*,*2*,*3* gene fusion testing, in which RNA-based NGS is regarded as the most comprehensive and generally preferred confirmatory method where feasible, whereas IHC, FISH, and RT-PCR are deployed as context-dependent fast-track screening or complementary methods [29,42]. RNA-based hybrid-capture NGS reliably detects both canonical and novel *NTRK1*,*2*,*3* gene rearrangements, circumventing many intronic breakpoint issues that undermine DNA-based approaches, and is explicitly recommended when broad coverage is required or when tumor histology does not suggest a restricted set of partners [42,62]. Within this methodical spectrum, our results support the potential of the ready-to-use EasyPGX ready *NTRK* fusion assay as a multiplex RT-PCR-based fast-track screening method to supplement routine tumor-agnostic *NTRK1*,*2*,*3* gene fusion detection strategies. The LOD of the assay, estimated at 6.25 ng RNA/reaction for detecting all tested *NTRK1*,*2*,*3* gene fusions and as low as 3.125 ng for individual *NTRK1*,*2*,*3* rearrangements (Table 2 and Figure 2), aligned with the RNA input ranges considered acceptable in formal RT-PCR validation guidance (e.g., AMP, Association for Molecular Pathology; CAP, College of American Pathologists). However, the inability of the assay to (i) distinguish several 5′ fusion partners and breakpoint combinations (summarized in Supplementary Table S1), and (ii) to detect novel *NTRK1*,*2*,*3* gene fusion partners or rare exon::exon breakpoint architectures not covered by its multiplex primer design represents a limitation inherent to any targeted RT-PCR primer design, which is addressed within multimodal algorithms by reflex confirmatory RNA-based NGS testing rather than by optimization of the streamlined ready-to-use laboratory workflow alone. Because current TRK inhibitor approvals are based on the presence of an *NTRK1*,*2*,*3* gene fusion irrespective of the specific 5′ partner, this limited partner resolution does not affect treatment eligibility, although partner identification may remain relevant for tumor classification and is provided by confirmatory RNA-based NGS.

The present study has several limitations that warrant careful consideration. First, although a broad range of *NTRK1*,*2*,*3* gene fusion variants and solid tumor types were included, the results may not be generalizable because of the retrospective, single-center design and modest sample size. The relatively wide 95% confidence intervals for *NTRK1*,*2*,*3* fusion transcripts represented by only one or a few fusion-positive samples reflect the limited cohort size and limited precision of the point estimate. Second, our institutional pathology cohort was enriched for *NTRK*-rearranged samples with specific exon::exon breakpoint architectures available in our institutional pathology archive, which might not cover the diverse spectrum of rare *NTRK1*,*2*,*3* gene fusion partners in other solid tumor cohorts. In addition, the enrichment for fusion-positive samples with known *NTRK1*,*2*,*3* rearrangement status may have favored cases with higher gene fusion transcript abundance, potentially overestimating assay sensitivity in an unselected prospective cohort. Additionally, the high prevalence of *NTRK*-rearranged samples in our institutional pathology cohort (55%, 16/29) inflates the PPV relative to real-world pan-cancer screening, where *NTRK1*,*2*,*3* gene fusion prevalence is <1% in most tumor types. In such low-prevalence scenarios, even a highly specific assay will yield a lower PPV, and negative results will have a higher confidence (NPV). Therefore, future studies in unselected cohorts are needed to estimate the PPV and NPV under clinically representative prevalence conditions. Third, RNA quality metrics (e.g., RNA integrity number, DV200) were not systematically available for this retrospective cohort; therefore, the impact of archival time and RNA integrity on the analytical performance of the assay (e.g., LOD, repeatability) was not evaluated. Consequently, the observation that FFPE blocks stored for up to six years yielded valid results should be interpreted with caution and cannot be generalized without prospective studies that systematically integrate RNA quality metrics. Nonetheless, the endogenous control met the predefined acceptance criteria in all reactions, indicating that sufficient amplifiable RNA was obtained from every tumor specimen irrespective of archival time. The estimated LOD (6.25 ng) may vary depending on RNA quality, and future studies should include RNA quality assessment to determine whether older or more degraded FFPE samples require higher RNA input quantities. Our serial dilution experiments to estimate the lowest input quantity of RNA used for qPCR amplification, generating the correct *NTRK1*,*2*,*3* gene fusion status, did not include the extensive replicate numbers and fusion-positive/fusion-negative sample mixture required to calculate a probabilistic LOD95. Our reproducibility assessment was limited to inter-run repeatability on a single EasyPGX qPCR instrument using the same kit reagent lot. Future studies should extend these analyses to include comprehensive fusion-positive/fusion-negative dilution series, multiple operators, different reagent lots, and additional diagnostic laboratories to comprehensively define the inter-laboratory reproducibility. Fourth, this study did not include a detailed comparison with alternative CE-IVD-compliant RT-PCR assays, IHC, or FISH workflows. Previous studies have reported pan-TRK IHC sensitivity ranging from 80% to 95% and specificity from 85% to 100%, with interpretive variability and false positives due to native TRK expression [43,44,45,46]. The indicated analytical performance (96% observed global sensitivity, 100% observed global specificity within its diagnostic coverage) appears competitive; however, future studies should compare the EasyPGX ready *NTRK* Fusion assay with alternative first-level fast-track platforms and screening methods to confirm its relative advantages in terms of cost-effectiveness, turnaround times, and analytical performance in routine multimodal pan-cancer diagnostic workflows.

Despite these limitations, in tumor types where chromosomal *NTRK1*,*2*,*3* rearrangements are relatively frequent and largely confined to a set of recurrent 5′ fusion partners (such as *ETV6::NTRK3*), the EasyPGX ready *NTRK* Fusion assay can serve as a first-level fast-track diagnostic workflow (approximately 3 h) that accelerates the identification of fusion-positive candidates for TRK inhibitor therapy and may accelerate the initial diagnosis of *NTRK* fusion-defined entities, particularly when NGS testing capacity or turnaround times are constrained. In common tumor types with very low (<1%) to low (1–5%) *NTRK1*,*2*,*3* gene fusion prevalence, current ESMO and international consensus recommendations highlight RNA-based NGS as the primary strategy, with IHC, FISH, or RT-PCR methods used selectively based on laboratory infrastructure and reimbursement [29,35,36,37,38,39]. Our results support such a pan-cancer diagnostic algorithm: the ready-to-use EasyPGX ready *NTRK* Fusion assay is best implemented as a first-level fast-track modality, supplementing multimodal tumor-agnostic testing workflows, and negative results should trigger confirmatory NGS-based reflex testing.

## 5. Conclusions

With the constraints of a single-center retrospective study, the CE-IVD-compliant EasyPGX ready *NTRK* Fusion assay demonstrated an observed overall sensitivity of 96% (23/24; 95% CI, 78.9–99.9%), with all fusion-positive transcripts detected for eight of nine tested gene fusion targets and 9/10 for *ETV6::NTRK3*. No false-positive results were observed for any of the nine fusion targets (observed specificity 100%; per-target 95% CIs are reported in Table 1). Within its predefined repertoire of tumor-agnostic *NTRK1*,*2*,*3* rearrangements, the assay achieved an estimated LOD of 6.25 ng RNA per reaction and conclusive results for all tumor specimens tested were generated in approximately 3 h. These findings support its potential as a first-level fast-track screening component of routine multimodal pan-cancer diagnostic workflows, with the understanding that negative results in high-suspicion cases require confirmatory RNA-based NGS reflex testing. We recommend multi-institutional validation to confirm the generalizability of our findings across diverse tumor types and clinical settings dedicated to precision oncology.

## Figures and Tables

**Table 1:** Analytic sensitivity and specificity of the EasyPGX ready *NTRK* Fusion assay for detecting predefined *NTRK1*,*2*,*3* gene fusion transcripts.

Gene Fusion	N° TP	N° FN	N° TN	N° FP	Sensitivity, SE % (95% CI)		Specificity, SP % (95% CI)	
*LMNA::NTRK1*	3	0	28	0	100.0	(29.2–100.0)	100.0	(87.7–100.0)
*TPM3::NTRK1*	4	0	27	0	100.0	(39.8–100.0)	100.0	(87.2–100.0)
*TPR::NTRK1*	1	0	30	0	100.0	(2.5–100.0)	100.0	(88.4–100.0)
*SQSTM1::NTRK1*	1	0	30	0	100.0	(2.5–100.0)	100.0	(88.4–100.0)
*TFG::NTRK1*	1	0	30	0	100.0	(2.5–100.0)	100.0	(88.4–100.0)
*AFAP1::NTRK2*	1	0	30	0	100.0	(2.5–100.0)	100.0	(88.4–100.0)
*QKI::NTRK2*	2	0	29	0	100.0	(15.8–100.0)	100.0	(88.1–100.0)
*TRIM24::NTRK2*	1	0	30	0	100.0	(2.5–100.0)	100.0	(88.4–100.0)
*ETV6::NTRK3*	9	1	24	0	90.0	(55.5–99.7)	100.0	(85.8–100.0)

TP, true positive; FN, false negative; TN, true negative; FP, false positive; CI, confidence interval. Sensitivity was calculated as TP/(TP + FN), and specificity as TN/(TN + FP). Two-sided 95% CIs were calculated using the exact Clopper–Pearson method for binomial proportions.

**Table 2:** Limit of detection (LOD) of the EasyPGX ready *NTRK* Fusion assay for specific *NTRK1*,*2*,*3* gene fusion transcripts.

		Limit of Detection (LOD)		
Gene Fusion	exon::exon	GDI Dx Archived FFPE Samples	Seraseq 0710-1031	Horizon HD834
*LMNA::NTRK1*	5::11	6.25 ng		
*LMNA::NTRK1*	6::11	6.25 ng		
*LMNA::NTRK1*	11::11		3.125 ng	
*TPM3::NTRK1*	8::10	6.25 ng	3.125 ng	3.125 ng
*TPR::NTRK1*	21::10	6.25 ng		
*SQSTM1::NTRK1*	6::10		3.125 ng	
*TFG::NTRK1*	5::10		3.125 ng	
*AFAP1::NTRK2*	14::12		3.125 ng	
*QKI::NTRK2*	6::16		6.25 ng	3.125 ng
*TRIM24::NTRK2*	12::15		3.125 ng	
*ETV6::NTRK3*	4::14		3.125 ng	
*ETV6::NTRK3*	5::14	6.25 ng	3.125 ng	
*ETV6::NTRK3*	4::15		3.125 ng	
*ETV6::NTRK3*	5::15	6.25 ng	3.125 ng	3.125 ng

## Data Availability

The original contributions presented in this study are included in the article/Supplementary Material. Further inquiries can be directed to the corresponding author.

## Supplementary Materials

The following supporting information can be downloaded at: https://www.mdpi.com/article/10.3390/cells15201833/s1. Supplementary Figure S1: Representative real-time RT-PCR amplification curves for the target (FAM) and endogenous control (HEX) channels. Exponential amplification plots generated by the EasyPGX qPCR instrument 96 for (A) *NTRK* fusion-positive, (B) *NTRK* fusion-negative, (C) by the EasyPGX ready *NTRK* Fusion multiplex primer design not detectable, and (D) discordant samples are shown. The red arrow marks a correct but weak target amplification fluorescence signal (FAM) above the predefined FAM Cq cutoff value of ≤35.0 for the *NTRK3* ex15 amplification mix. In the presence of an *NTRK* gene fusion, the target-specific probes anneal and emit fluorescence, generating an exponential amplification curve that crosses the threshold line. If only an amplification curve appears for the endogenous control (HEX), it indicates that (i) sufficient RNA sample quality is verified, (ii) no RT-PCR inhibitors are present, and (iii) no *NTRK* gene fusion transcripts are detectable. Supplementary Table S1: Molecular details of *NTRK1*, *NTRK2*, and *NTRK3* gene fusions theoretically detectable by the EasyPGX ready *NTRK* fusion kit. According to kit user manual Appendix A: EasyPGX ready *NTRK* Fusion User Manual (RT035, version 2022/03, https://www.diatechpharmacogenetics.com/; accessed on 31 August 2026).

## Abbreviations

BRAF	B-raf proto-oncogene
CE-IVD	European Conformity—In Vitro Diagnostic
CI	Confidence interval
DNA	Deoxyribonucleic acid
FFPE	Formalin-fixed, paraffin-embedded
FISH	Fluorescence in situ hybridization
FN	False negative
FP	False positive
HER2	Human epidermal growth factor receptor 2
IHC	Immunohistochemistry
LOD	Limit of detection
MSI-H	Microsatellite instability-high
NGS	Next-generation sequencing
NPV	Negative predictive value
NTRK	Neurotrophic tropomyosin receptor kinase
PPV	Positive predictive value
qPCR	Quantitative polymerase chain reaction
RET	Rearranged during transfection
RNA	Ribonucleic acid
RT-PCR	Reverse transcription polymerase chain reaction
SE	Sensitivity
SP	Specificity
TMB-H	Tumor mutational burden-high
TN	True negative
TP	True positive
TRK	Tropomyosin receptor kinase
WT	Wild-type
