# Supplementary material for: Analytical Performance of the EasyPGX Ready NTRK Fusion Assay for Detecting Tumor-Agnostic NTRK Gene Fusions in Solid Tumors
Source: Cells. 2026 Oct 9;15(20):1833. doi: 10.3390/cells15201833 (PMC13702506; doi:10.3390/cells15201833)
Supplement: Supplementary file 1 [file cells-15-01833-s001.zip › 2026_MDPI_Cells_SoironA_EasyPGX_NTRK_SupFigureS1.pdf]

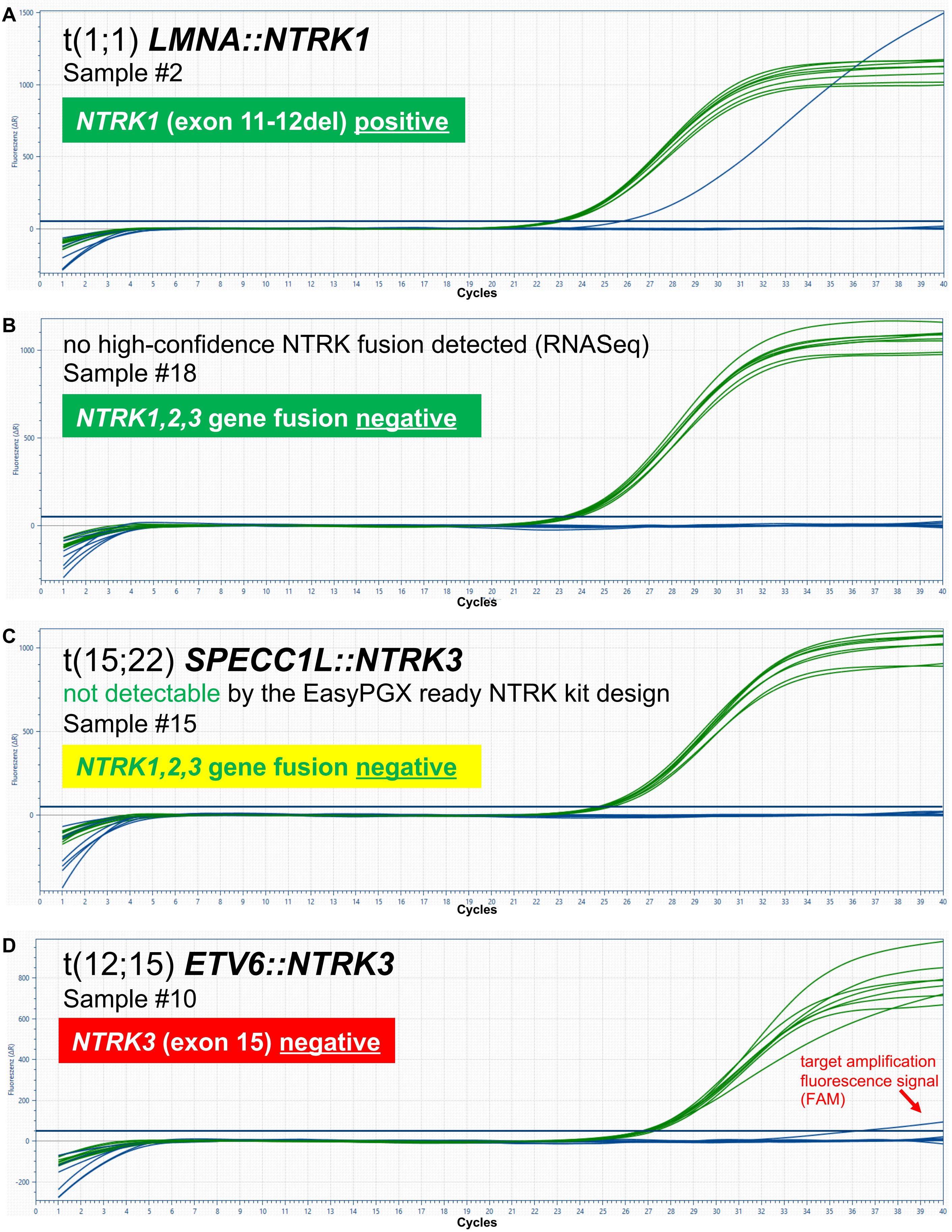

Supplementary Figure S1: Representative real-time RT-PCR amplification curves for the target (FAM) and endogenous control (HEX) channels. Exponential amplification plots generated by the EasyPGX qPCR instrument 96 for **(A)** *NTRK* fusion-positive, **(B)** *NTRK* fusion-negative, **(C)** by the EasyPGX ready *NTRK* Fusion multiplex primer design not detectable, and **(D)** discordant samples are shown. The red arrow marks a correct but weak target amplification fluorescence signal (FAM) above the predefined FAM Cq cut-off value of ≤ 35.0 for the *NTRK3* ex15 amplification mix. In the presence of an *NTRK* gene fusion, the target-specific probes anneal and emit fluorescence, generating an exponential amplification curve that crosses the threshold line. If only an amplification curve appears for the endogenous control (HEX), it indicates that (i) sufficient RNA sample quality is verified, (ii) no RT-PCR inhibitors are present, and (iii) no *NTRK* gene fusion transcripts are detectable.
