# Supplementary material for: Analytical Performance of the EasyPGX Ready NTRK Fusion Assay for Detecting Tumor-Agnostic NTRK Gene Fusions in Solid Tumors
Source: Cells. 2026 Oct 9;15(20):1833. doi: 10.3390/cells15201833 (PMC13702506; doi:10.3390/cells15201833)
Supplement: Supplementary file 1 [file cells-15-01833-s001.zip › 2026_MDPI_Cells_SoironA_EasyPGX_NTRK_SupTableS1.pdf]

Supplementary Table S1

Molecular details of *NTRK1, NTRK2,* and *NTRK3* gene fusions theoretically detectable by the EasyPGX ready *NTRK* fusion kit.
According to Appendix A: EasyPGX ready *NTRK* Fusion User Manual (RT035, version 2022/03, https://www.diatechpharmacogenetics.com/)

Fusion	Structure	Reference
**NTRK1 exon 9-10 (not distinguishable between them)**		
TFG-NTRK1 T5:N9	TFG{ENST00000240851}:r.1_920_NTRK1{ENST00000392302}:r.935_2609	Greco A et al. 1995. (PMID: 7565764)
NFASC-NTRK1 N20:N10	NFASC{ENST00000360049}:r.1_2786_NTRK1{ENST00000392302}:r.1262_2609	Kim J et al 2014. (PMID: 24647444)
NFASC-NTRK1 N21:N10	NFASC{ENST00000360049}:r.1_2902_NTRK1{ENST00000392302}:r.1262_2609	Kim J et al 2014. (PMID: 24647444)
**NTRK1 exon 10 (not distinguishable between them)**		
SQSTM1-NTRK1 S4:N10	SQSTM1{ENST00000360718}:r.1_1022_NTRK1{ENST00000392302}:r.1262_2609	Farago AF et al 2015. (PMID: 26565381)
TFG-NTRK1 T5:N10	TFG{ENST00000240851}:r.1_920_NTRK1{ENST00000392302}:r.1262_2609	Greco A et al. 1995. (PMID: 7565764)
TPM3-NTRK1 T7:N10	TPM3{ENST00000368533}:r.1_717_NTRK1{ENST00000392302}:r.1262_2609	Ardini E et al 2014. (PMID: 24962792)
TPR-NTRK1 T21:N10	TPR{ENST00000367478}:r.1_3073_NTRK1{ENST00000392302}:r.1262_2609	Greco A et al 1997. (PMID: 9172002)
TRIM63-NTRK1 T8:N10	TRIM63{ENST00000374272}:r.1_1190_NTRK1{ENST00000392302}:r.1262_2609	Hechtman JF et al 2018. (PMID: 28719467)
**NTRK1 exon 11-12del (not distinguishable between them)**		
BCAN-NTRK1 B13:N11	BCAN{ENST00000329117}:r.1_2964_NTRK1{ENST00000392302}:r.1318_2609	Kim J et al 2014. (PMID: 24647444)
LMNA-NTRK1 L11del178:N11	LMNA{ENST00000368300}:r.1_2030_NTRK1{ENST00000392302}:r.1318_2609	Russo M et al 2016. (PMID: 26546295)
LMNA-NTRK1 L2:N11	LMNA{ENST00000368300}:r.1_725_NTRK1{ENST00000392302}:r.1318_2609	Wiesner T et al 2014. (PMID: 24445538)
PPL-NTRK1 P22del2181:N11	PPL{ENST00000345988}:r.1_3180_NTRK1{ENST00000392302}:r.1318_2609	Zheng Z et al 2014. (PMID: 25384085)
TPR-NTRK1 T6del122:N12del99	TPR{ENST00000367478}:r.1_871_NTRK1{ENST00000392302}:r.1520_2609	Greco A et al 1997. (PMID: 9172002)
**NTRK1 exon 12 (not distinguishable between them)**		
MPRIP-NTRK1 M21:N12	MPRIP{ENST00000395811}:r.1_3048_NTRK1{ENST00000392302}:r.1421_2609	Vaishnavi A et al 2013. (PMID: 24162815)
MPRIP-NTRK1 M18:N12	MPRIP{ENST00000395811}:r.1_2678_NTRK1{ENST00000392302}:r.1421_2609	Vaishnavi A et al 2013. (PMID: 24162815)
MPRIP-NTRK1 M14:N12	MPRIP{ENST00000395811}:r.1_2135_NTRK1{ENST00000392302}:r.1421_2609	Vaishnavi A et al 2013. (PMID: 24162815)
SCYL3-NTRK1 S11:N12	SCYL3{ENST00000367771}:r.1_1527_NTRK1{ENST00000392302}:r.1421_2609	Milione M et al 2014. (PMID: 28903424)
TPM3-NTRK1 T7:N12	TPM3{ENST00000368533}:r.1_717_NTRK1{ENST00000392302}:r.1421_2609	Farago AF et al 2015. (PMID: 26565381)
TPR-NTRK1 T6:N12	TPR{ENST00000367478}:r.1_993_NTRK1{ENST00000392302}:r.1421_2609	Greco A et al 1997. (PMID: 9172002)
**NTRK2 exon 12-15 (not distinguishable between them)**		
AFAP1-NTRK2 A13:N12	AFAP1{ENST00000360265}:r.1_2056_NTRK2{ENST00000376214}:r.2098_5608	Stransky N et al 2014. (PMID: 25204415)
VCL-NTRK2 V16:N12	VCL{ENST00000211998}:r.1_2528_NTRK2{ENST00000376214}:r.2098_5608	Wu G et al 2014. (PMID: 24705251)
TLE4-NTRK2 T7:N15	TLE4{ENST00000265284}:r.1_580_NTRK2{ENST00000376214}:r.2335_5608	Prabhakaran N et al 2018. (PMID: 29502353)
TRIM24-NTRK2 T12:N15	TRIM24{ENST00000343526}:r.1_2229_NTRK2{ENST00000376214}:r.2335_5608	Stransky N et al 2014. (PMID: 25204415)
**NTRK2 exon 16-17 (not distinguishable between them)**		
AGBL4-NTRK2 A6:N16	AGBL4{ENST00000371839}:r.1_792_NTRK2{ENST00000376214}:r.2397_5608	Wu G et al 2014. (PMID: 24705251)
QKI-NTRK2 Q6:N16	QKI{ENST00000361752}:r.1_1485_NTRK2{ENST00000376214}:r.2397_5608	Ni J et al 2017. (PMID: 27402815)
SQSTM1-NTRK2 S4:N16	SQSTM1{ENST00000360718}:r.1_1022_NTRK2{ENST00000376214}:r.2397_5608	Stransky N et al 2014. (PMID: 25204415)
STRN-NTRK2 S3:N16	STRN{ENST00000263918}:r.1_421_NTRK2{ENST00000376214}:r.2397_5608	Wu LW et al 2018. (doi:10.1200/po.18.00101)
SQSTM1-NTRK2 S4:N17	SQSTM1{ENST00000360718}:r.1_1022_NTRK2{ENST00000376214}:r.2572_5608	Stransky N et al 2014. (PMID: 25204415)
**NTRK3 exon 14 (not distinguishable between them)**		
ETV6-NTRK3 E4:N14	ETV6{ENST00000396373}:r.1_737_NTRK3{ENST00000360948}:r.1703_3004	Leeman-Neill RJ et al 2014. (PMID: 24327398)
ETV6-NTRK3 E5:N14	ETV6{ENST00000396373}:r.1_1283_NTRK3{ENST00000360948}:r.1703_3004	Leeman-Neill RJ et al 2014. (PMID: 24327398)
**NTRK3 exon 15 (not distinguishable between them)**		
ETV6-NTRK3 E4:N15	ETV6{ENST00000396373}:r.1_737_NTRK3{ENST00000360948}:r.1904_3004	Eguchi M et al 1999. (PMID: 9949179)
ETV6-NTRK3 E5:N15	ETV6{ENST00000396373}:r.1_1283_NTRK3{ENST00000360948}:r.1904_3004	Sheng WQ et al 2001. (PMID: 11242790)
